# Supplementary material for: Software engineering principles to improve quality and performance of R software
Source: PeerJ Comput Sci. 2019 Feb 4;5:e175. doi: 10.7717/peerj-cs.175 (PMC7924430; doi:10.7717/peerj-cs.175)
Supplement: Supplemental Information 1 — “All” column summarizes data from years from to 2005 up through 2018. [file peerj-cs-05-175-s001.docx]

## SUPPLEMENT TABLE S1

For data tables, “All” column summarizes data from years from to 2005 up through 2018.

Table for data shown in Figure 1: Packages by year updated and presence of non-empty testing directory

| Year | 2008 | 2009 | 2010 | 2011 | 2012 | 2013 | 2014 | 2015 | 2016 | 2017 | 2018 | All |
| --- | --- | --- | --- | --- | --- | --- | --- | --- | --- | --- | --- | --- |
| Packages | 10 | 24 | 32 | 65 | 457 | 564 | 755 | 1127 | 1726 | 2517 | 6226 | 13509 |
| Pkg w/Test Dir | 2 | 5 | 4 | 10 | 33 | 64 | 91 | 212 | 467 | 863 | 2722 | 4474 |
| Tested % | 20 | 21 | 12 | 15 | 7 | 11 | 12 | 19 | 27 | 34 | 44 | 33 |
