# Supplementary material for: Software engineering principles to improve quality and performance of R software
Source: PeerJ Comput Sci. 2019 Feb 4;5:e175. doi: 10.7717/peerj-cs.175 (PMC7924430; doi:10.7717/peerj-cs.175)
Supplement: Supplemental Information 2 — Only non-zero percentages shown; “All” column summarizes data from years from to 2005 up through 2018. [file peerj-cs-05-175-s002.docx]

## SUPPLEMENT TABLE S2

For data tables, “All” column summarizes data from years from to 2005 up through 2018.

Table for data shown in Figure 2: Packages by year updated and testing framework dependency; only non-zero percentages shown. “Packages” column shows count of all packages with no dependency plus the sum of all separate dependencies; packages with multiple dependencies are counted multiple times.

| Year | 2008 | 2009 | 2010 | 2011 | 2012 | 2013 | 2014 | 2015 | 2016 | 2017 | 2018 | All |
| --- | --- | --- | --- | --- | --- | --- | --- | --- | --- | --- | --- | --- |
| Packages | 10 | 24 | 32 | 65 | 457 | 564 | 755 | 1130 | 1729 | 2518 | 6235 | 13525 |
| Pkgs w/Dep | 0 | 0 | 0 | 2 | 5 | 22 | 30 | 129 | 373 | 705 | 2340 | 3606 |
| svUnit | 0 | 0 | 0 | 0 | 1 | 0 | 0 | 0 | 2 | 1 | 5 | 9 |
| RUnit (%) | 0 | 0 | 0 | 1 (2) | 3 (1) | 1 | 6 (1) | 9 (1) | 8 | 31 (1) | 72 (1) | 131 |
| testthat (%) | 0 | 0 | 0 | 1 (2) | 1 | 21 (4) | 24 (3) | 118 (10) | 361 (21) | 668 (27) | 2243 (36) | 3437 |
| testit | 0 | 0 | 0 | 0 | 0 | 0 | 0 | 2 | 2 | 3 | 18 | 25 |
| unitizer | 0 | 0 | 0 | 0 | 0 | 0 | 0 | 0 | 0 | 1 | 2 | 3 |
| unittest | 0 | 0 | 0 | 0 | 0 | 0 | 0 | 0 | 0 | 1 | 0 | 1 |
