# Supplementary material for: Software engineering principles to improve quality and performance of R software
Source: PeerJ Comput Sci. 2019 Feb 4;5:e175. doi: 10.7717/peerj-cs.175 (PMC7924430; doi:10.7717/peerj-cs.175)
Supplement: Supplemental Information 3 — “All” column summarizes data from years from to 2005 up through 2018. [file peerj-cs-05-175-s003.docx]

## SUPPLEMENT TABLE S3

For data tables, “All” column summarizes data from years from to 2005 up through 2018.

Table for data shown in Figure 3: Packages by year updated and presence of non-empty src directory

| Year | 2008 | 2009 | 2010 | 2011 | 2012 | 2013 | 2014 | 2015 | 2016 | 2017 | 2018 | All |
| --- | --- | --- | --- | --- | --- | --- | --- | --- | --- | --- | --- | --- |
| Packages | 10 | 24 | 32 | 65 | 457 | 564 | 755 | 1127 | 1726 | 2517 | 6226 | 13509 |
| Pkg w/Src | 1 | 6 | 11 | 15 | 59 | 114 | 154 | 224 | 376 | 606 | 1859 | 3428 |
| Src % | 10 | 25 | 34 | 23 | 13 | 20 | 20 | 20 | 22 | 24 | 30 | 25 |
