# Supplementary material for: Software engineering principles to improve quality and performance of R software
Source: PeerJ Comput Sci. 2019 Feb 4;5:e175. doi: 10.7717/peerj-cs.175 (PMC7924430; doi:10.7717/peerj-cs.175)
Supplement: Supplemental Information 4 — Only non-zero percentages shown; “All” column summarizes data from years from to 2005 up through 2018. [file peerj-cs-05-175-s004.docx]

**SUPPLEMENT TABLE S4**

For data tables, “All” column summarizes data from years from to 2005 up through 2018.

Table for data shown in Figure 4: Packages by year updated and optimization framework dependency; only non-zero percentages shown. “Packages” column shows count of all packages with no dependency plus the sum of all separate dependencies; packages with multiple dependencies are counted multiple times.

| Year | 2009 | 2010 | 2011 | 2012 | 2013 | 2014 | 2015 | 2016 | 2017 | 2018 | All |
| --- | --- | --- | --- | --- | --- | --- | --- | --- | --- | --- | --- |
| Packages | 24 | 32 | 66 | 463 | 566 | 773 | 1170 | 1822 | 2699 | 6964 | 14595 |
| Packages w/Dep | 0 | 1 | 2 | 18 | 28 | 95 | 176 | 367 | 641 | 2368 | 3696 |
| Rcpp | 0 | 1 (3) | 0 | 4 (1) | 6 (1) | 37 (5) | 60 (5) | 149 (8) | 273 (10) | 998 (14) | 1528 |
| tictoc | 0 | 0 | 0 | 0 | 0 | 0 | 0 | 0 | 1 | 4 | 5 |
| rbenchmark | 0 | 0 | 0 | 0 | 0 | 0 | 1 | 1 | 3 | 16 | 21 |
| microbenchmark | 0 | 0 | 0 | 0 | 0 | 0 | 0 | 5 | 11 | 67 (1) | 83 |
| benchr | 0 | 0 | 0 | 0 | 0 | 0 | 0 | 0 | 0 | 1 | 1 |
| profr | 0 | 0 | 0 | 0 | 0 | 0 | 0 | 1 | 0 | 1 | 2 |
| profvis | 0 | 0 | 0 | 0 | 0 | 0 | 0 | 0 | 0 | 1 | 1 |
| snow | 0 | 0 | 0 | 4 (1) | 1 | 0 | 1 | 0 | 5 | 10 | 21 |
| doSNOW | 0 | 0 | 0 | 1 | 0 | 2 | 0 | 0 | 4 | 9 | 16 |
| parallel | 0 | 0 | 0 | 3 (1) | 14 (2) | 32 (4) | 53 (5) | 102 (6) | 169 (6) | 568 (8) | 941 |
| doParallel | 0 | 0 | 0 | 0 | 1 | 4 (1) | 20 (2) | 38 (2) | 68 (3) | 259 (4) | 390 |
| Rmpi | 0 | 0 | 0 | 2 | 0 | 0 | 7 (1) | 0 | 4 | 10 | 23 |
| foreach | 0 | 0 | 1 (2) | 3 (1) | 5 (1) | 19 (2) | 30 (3) | 61 (3) | 83 (3) | 305 (4) | 507 |
| future | 0 | 0 | 0 | 0 | 0 | 0 | 0 | 0 | 4 | 35 (1) | 39 |
| future.apply | 0 | 0 | 0 | 0 | 0 | 0 | 0 | 0 | 0 | 16 | 16 |
| SparkR | 0 | 0 | 0 | 0 | 0 | 0 | 0 | 0 | 0 | 1 | 1 |
| sparklyr | 0 | 0 | 0 | 0 | 0 | 0 | 0 | 1 | 1 | 9 | 11 |
| batchtools | 0 | 0 | 0 | 0 | 0 | 0 | 0 | 0 | 0 | 3 | 3 |
| RcppParallel | 0 | 0 | 0 | 0 | 0 | 0 | 0 | 2 | 6 | 27 | 35 |
| parallelDist | 0 | 0 | 0 | 0 | 0 | 0 | 0 | 0 | 0 | 5 | 5 |
| parallelMap | 0 | 0 | 0 | 0 | 0 | 0 | 0 | 2 | 4 | 8 | 14 |
| doMC | 0 | 0 | 1 (2) | 1 | 1 | 1 | 3 | 5 | 4 | 10 | 26 |
| doMPI | 0 | 0 | 0 | 0 | 0 | 0 | 0 | 0 | 0 | 5 | 5 |
| partools | 0 | 0 | 0 | 0 | 0 | 0 | 0 | 0 | 1 | 0 | 1 |
| DSL | 0 | 0 | 0 | 0 | 0 | 0 | 1 | 0 | 0 | 0 | 1 |
